# Supplementary figures and images for: Chromatin interaction maps identify Wnt responsive cis-regulatory elements coordinating Paupar-Pax6 expression in neuronal cells
Source: PLoS Genet. 2022 Jun 16;18(6):e1010230. doi: 10.1371/journal.pgen.1010230 (PMC9202886; doi:10.1371/journal.pgen.1010230)

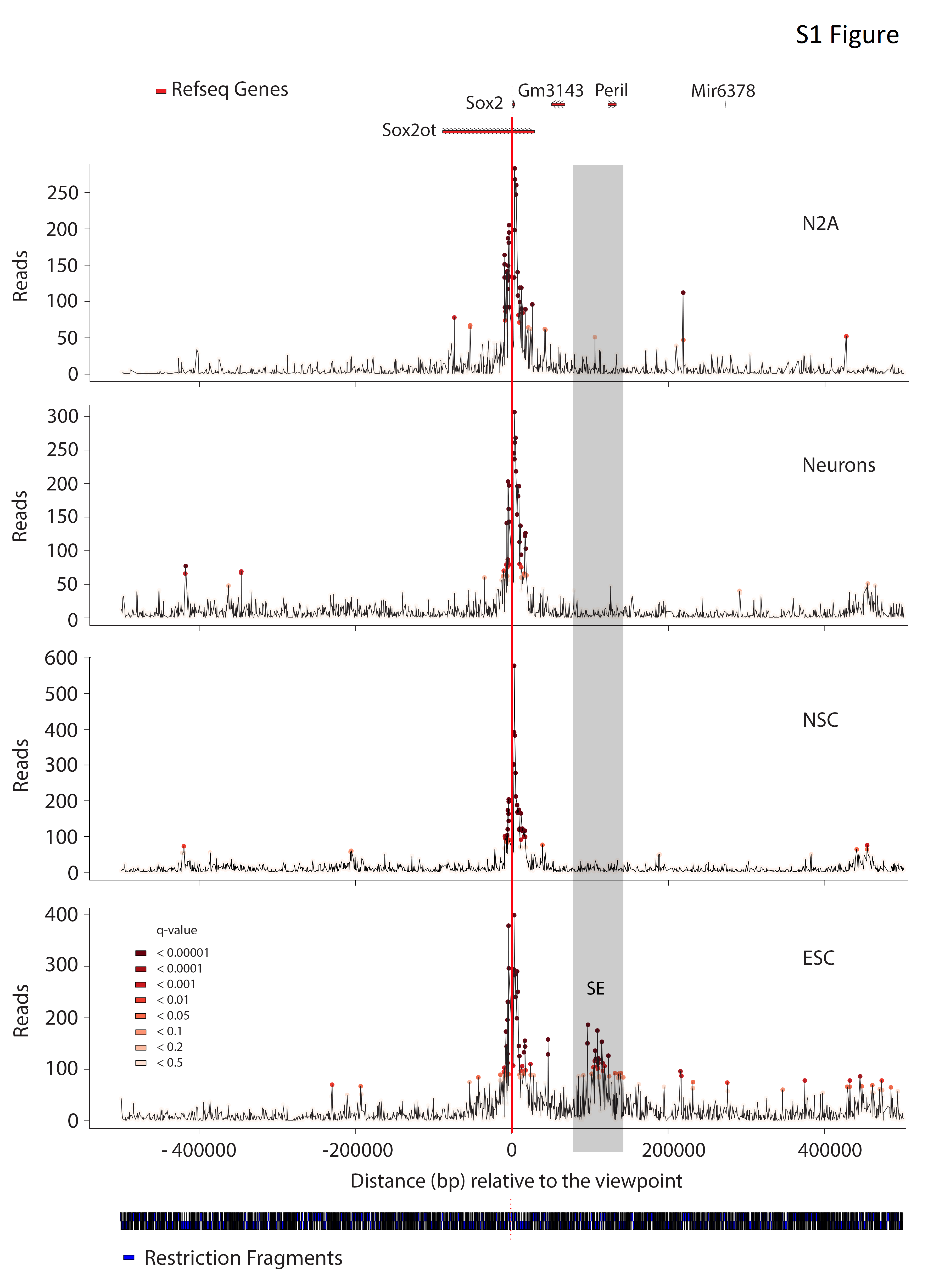

Supplement: S1 Fig — NG Capture-C profiles displaying the Sox2 promoter interaction count per DpnII restriction enzyme fragment in the indicated cell types. The red vertical line indicates the location of the Sox2 promoter viewpoint. Significant interactions were determined using r3C-seq. (TIF) [file pgen.1010230.s001.tif]

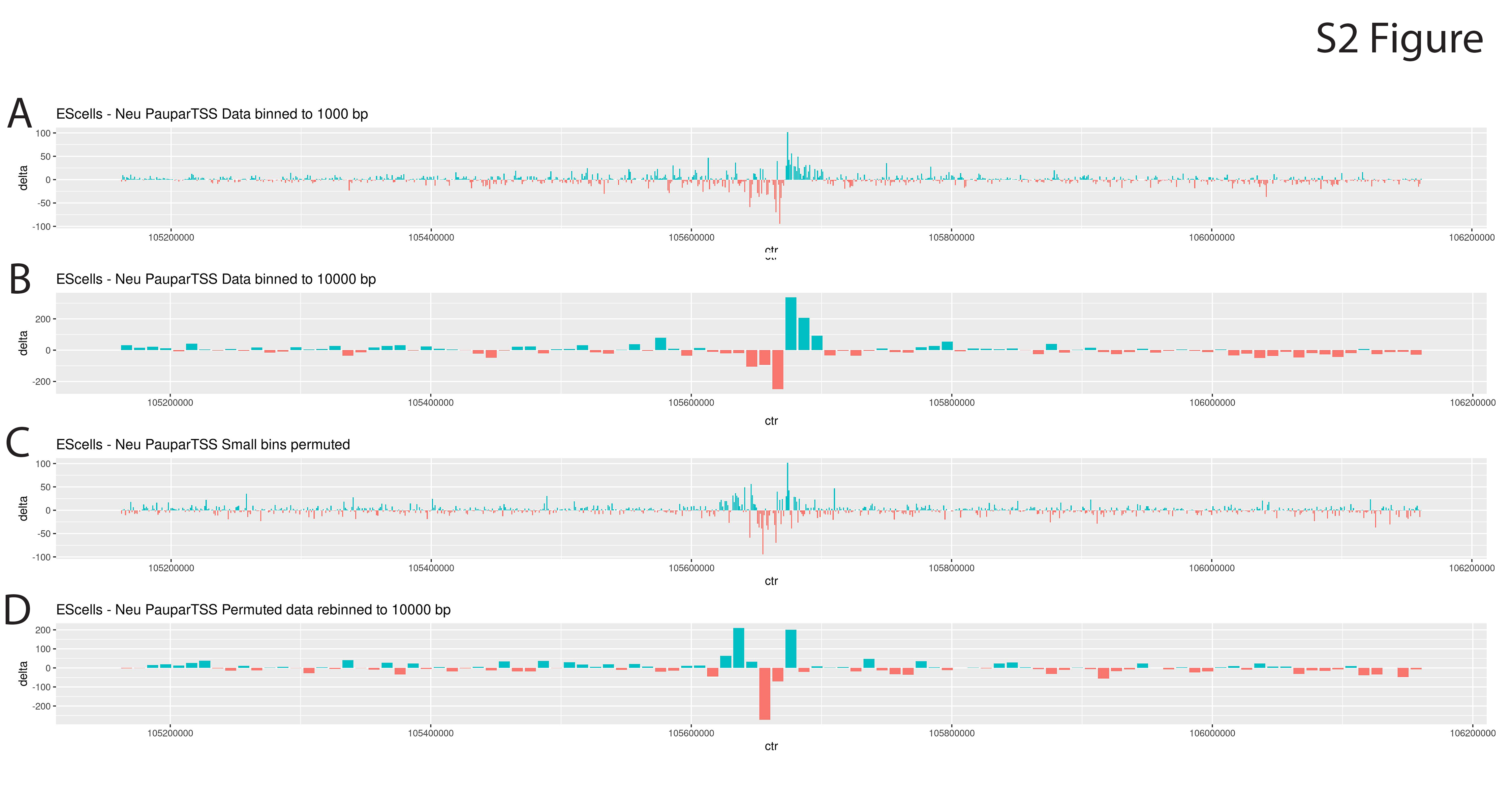

Supplement: S2 Fig — Differences in mean normalized NG Capture-C counts between neurons and ESCs for interactions with the Paupar viewpoint are plotted on the y-axis. X-axis shows position on chromosome 2 (GRCm38/mm10). Sequence data was permuted to assess specificity and statistical significance was calculated as described in Materials and Methods. (A) Difference in mean normalised interactions between ESCs and neurons binned to 1 kb. Negative values shown in red indicate increased interactions in neurons. Positive values in turquoise illustrate increased interactions in ESCs. (B) The same data binned to 10 kb. Note the emergence of the large red region approximately 350 kb downstream of Pax6 and the asymmetric pattern near the viewpoint. (C) The 1 kb bins from (A) permuted. Bins further than 50 kb from the viewpoint are permuted at random. Bins closer than 50 kb are only permuted keeping their distance from the viewpoint. (D) The previous panel re-binned to 10kb. Notice that no large contiguous regions of constant sign appear, nor does the asymmetric pattern seen near the viewpoint in (B). (TIF) [file pgen.1010230.s002.tif]

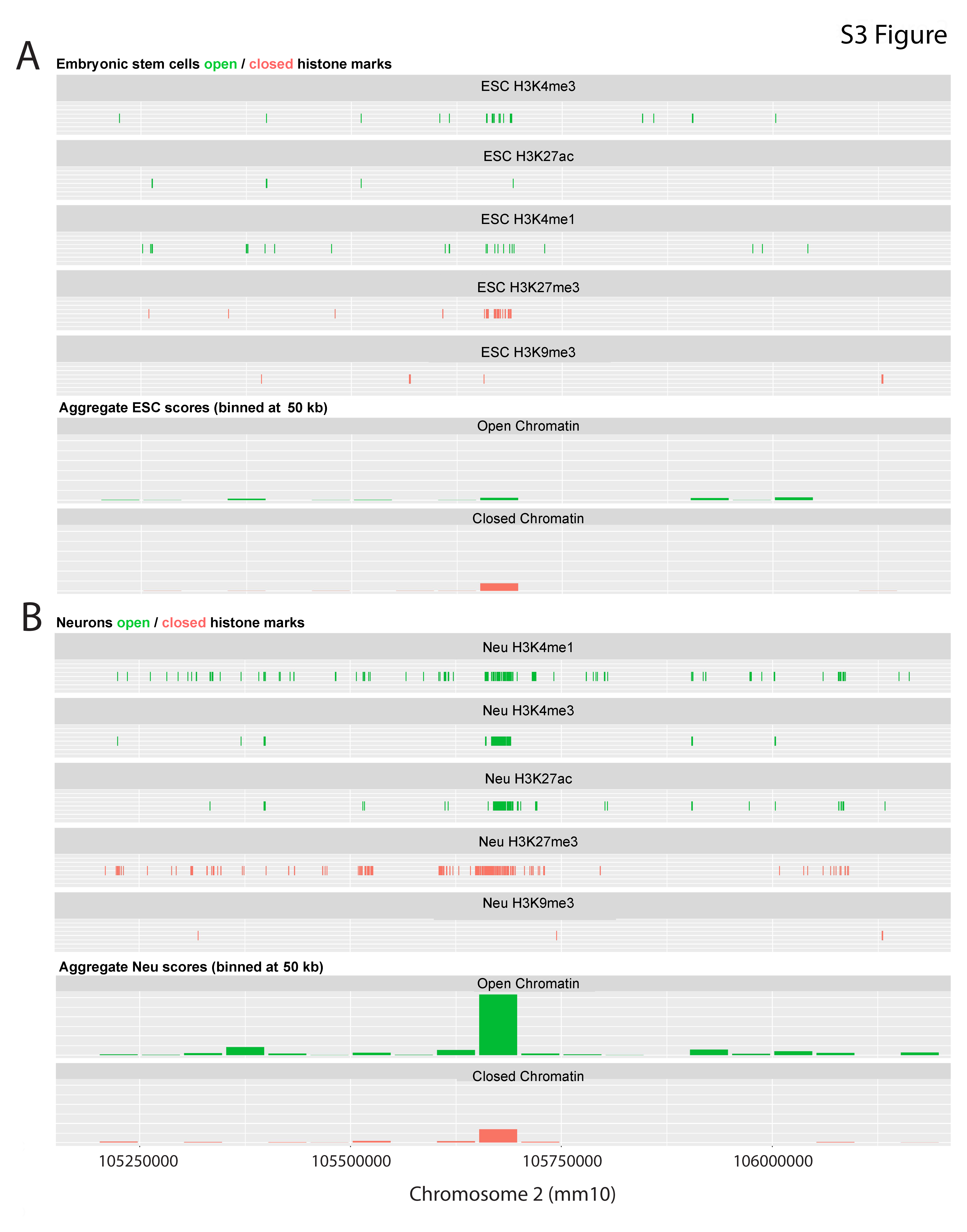

Supplement: S3 Fig — ENCODE Project ChIP-seq data mapping the location of open (H3K4me1, H3K4me3 and H3K27ac) and closed (H3K27me3 and H3K9me3) ChIP-seq peaks in ESCs (A) and E12.5 mouse forebrain tissue (B) across approximately 1MB genomic sequence surrounding the Paupar-Pax6 locus [24]. Individual peaks of less than 1000 bp are shown at 1000 bp long for visibility reasons. Aggregated data represent summed and binned scores from the individual tracks. (TIF) [file pgen.1010230.s003.tif]
