# Supplementary material for: Chromatin interaction maps identify Wnt responsive cis-regulatory elements coordinating Paupar-Pax6 expression in neuronal cells
Source: PLoS Genet. 2022 Jun 16;18(6):e1010230. doi: 10.1371/journal.pgen.1010230 (PMC9202886; doi:10.1371/journal.pgen.1010230)
Supplement: Attachment — Submitted filename: PLosGeneticsReviewsResponseFinal.docx [file pgen.1010230.s013.docx]

Dear Dr. Vance,

Thank you very much for submitting your manuscript "Chromatin interaction maps identify Wnt responsive cis-regulatory elements coordinating Paupar-Pax6 expression in neuronal cells" (PGENETICS-D-21-00785) for consideration at PLOS Genetics.

The manuscript was fully evaluated at the editorial level and by independent peer reviewers. As you will see, the reviewers identified a number of serious concerns. Based on the reviews and our editorial evaluation, we regret to say that we will be unable to consider the manuscript further at PLOS Genetics.

The consensus is that there are weaknesses in experimental design and statistical analysis supporting the main conclusions and that additional work that provides more biological evidence and rigor of statistical analysis would be necessary for PLOS Genetics. The editors would be willing to consider a future new submission of the manuscript in which the current work serves as a starting point, and addresses the major concerns raised by the reviewers (including a better description of the motifs analysis, genomics to better assess TCF7L2 genome-wide binding, controls in the dCas9 experiment, and statistical analysis to support some of the major claims) and clarifying inconsistencies with previous studies.

The reviews are attached below this email, and we hope you will find them helpful if you decide to revise the manuscript for submission elsewhere. We are sorry that we cannot be more positive on this occasion. We very much appreciate your wish to present your work in one of PLOS's Open Access publications.

Thank you for your support, and we hope that you will consider PLOS Genetics for other submissions in the future.

Sincerely,

Ivan D'Orso, PhD

Guest Editor

PLOS Genetics

John Greally

Section Editor: Epigenetics

PLOS Genetics

**************************************

Thank you for considering this improved version of our manuscript. We now submit a substantially improved version containing new data and analysis all of which support and strengthen our original claims. This includes:

(1) Statistical analysis identifying significant overlap between H3K4me1 ChIP-seq peaks in mouse forebrain tissue (but not in ESCs) and chromatin interactions with the Pax6 promoters.

(2) More detail on the motif analysis and extra esiRNA knockdown experiments. These define four other neuronal transcription factors (Ascl1, Klf16, Sox6, Arid3a), in addition to TCF7L2, that have multiple motifs (>10) within candidate CREs and regulate either *Paupar* or *Pax6* expression in N2A cells.

(3) We present a more comprehensive set of CRISPRi experiments in which we target all four ChIP-defined TCF7L2 bound candidate CREs (CRE1, CRE2, CRE3, CRE5) as well as two nearby non-TCF7L2 motif containing control sequences within the *Paupar*-*Pax6* regulatory region. We found that CRE2 and CRE5 are transcriptional repressors while CRE3 is an enhancer, whilst dCas9-KRAB recruitment to CRE1 and the control regions did not lead to coordinated changes in *Paupar-Pax6* expression. These additional data confirm the sequence specificity of the CRE2, CRE3 and CRE5 transcriptional response.

(4) We performed reporter experiments showing that Paupar transcript can activate Pax6. We use this data to reconcile some differences with our published work and highlight distinct functions for *Paupar* transcript and its DNA locus.

(5) We showed that Tcf7L2, Paupar and Pax6 levels are reduced following expression of constitutively active Bcat S33Y and present extra data showing that Rspo2 treatment does not change Tcf7L2, Paupar and Pax6. Together, the pathway manipulation and Tcf7L2 knockdown experiments all show that any changes in Tcf7L2 expression lead to reciprocal changes in Paupar-Pax6. This provides more evidence that Wnt works through TCF7L2 to coordinate Paupar-Pax6 co-expression.

Please find a detailed a point-by-point response to the reviewers’ comments below.

Reviewer's Responses to Questions

Reviewer #1: In this manuscript, Pavlaki et al performed experiments to probe the regulatory architecture of the Pax6 transcription factor and the nearby Paupar lncRNA. The authors first performed NG Capture-C using multiple probes directed against promoters of Paupar and Pax6 in ESCs, NCS, Neurons, and N2A cells. They identified interacting regions for all probed regions in all cell types, though there appear to be similar across cell types. The authors then performed perturbation experiments to show that the Wnt pathway regulates Paupar and Pax6 expression. The authors then show by ChIP that Tcf7l2 binds to cis-regulatory elements in the locus, and performed CRISPRi experiments to suggest that several CREs are silencers and promoters. Finally, the authors performed additional statistical analysis to examine interaction asymmetry and to identify new genomic regions that potentially regulate Pax6/Paupar.

In this reviewer’s opinion, the strengths of this manuscript are the perturbation experiments in Figure 4, which clearly illustrate the coordinate regulation of Paupar and Pax6. However, some inconsistencies in the results section and with published work are confusing (see specific points below). There are two central weaknesses of this manuscript: the lack of statistical evidence for some of the claims and the lack of independent experimental evidence for genomic observations (see below). Overall, the conclusions of this manuscript are only somewhat supported by the data. The inconsistencies need to be resolved or clarified.

We thank the reviewer for recognising the importance of the perturbation experiments that “clearly illustrate the coordinate regulation of Paupar and Pax6”.

Specific points:

1. The authors write: “chromatin interactions with the Paupar and Pax6 promoters show an increased overlap with H3K4me1 ChIP-seq peaks in E12.5 mouse forebrain tissue compared to ESCs using publicly available ENCODE data”. This is not clear from the data presented, and statistical evidence is needed.

As requested, we evaluated the statistical significance in the overlap between H3K4me1 ChIP-seq peaks (in mouse forebrain tissue and ESCs) and chromatin interactions with the Paupar and Pax6 promoters.

To do this, we first identified H3K4me1 peaks within 50 kb up- or down-stream of the midpoint of each viewpoint using publicly available E12.5 mouse forebrain tissue and ESCs ChIP-seq data. For each of the viewpoints and cell types we counted the number of overlaps between the H3K4me1 peaks and the duplicated chromatin interactions. We then performed permutation testing in the following manner. For each viewpoint and cell type we performed 100 trials. In each trial, the locations of the H3K4me1 peaks within the region of interest were randomly permuted and the number of peaks overlapping duplicated chromatin interactions were counted. We then compared the actual number of overlaps to the numbers of overlaps in the 100 randomised trials. Discovering that the real count is larger than all but 5 of the randomised counts detects enrichment with a p-value of 0.05 or less.

This approach revealed statistically significant enrichment (p<0.05) in the overlap between mouse forebrain H3K4me1 ChIP-seq peaks and chromatin interactions with the Pax6 P0 and P1 promoters. We also observed enrichment in the case of the *Paupar* promoter viewpoint, but not at a statistically significant level. We did not detect significant enrichment (p<0.05) between ESC H3K4me1 peaks and chromatin interactions with any promoter viewpoint. As H3K4me1 marks both active and poised enhancers this implies that the NG Capture-C interactions may function to control expression of the *Paupar-Pax6* locus in neuronal cells.

We have modified the text in the results section accordingly to highlight the new statistical evidence supporting our conclusions and the permutation testing counts are shown in a new Supplemental Table S4.

2. The authors write: “most curated fragments are located at curvature points on the chromatin fibre and appear to be orientated towards the Paupar-Pax6 promoters”. This seems like a subjective interpretation, and is not clear from the data presented. Also, it is hard to get statistical significance from computational visualizations of 3D looping. Can the authors reframe the computational analysis in a way that can yield more statistical evidence?

We used the 4Cin computational pipeline to generate a 3D model of *Paupar*-*Pax6* local chromatin architecture from NG Capture-C data and visualise the relative proximity of the curated fragments to the *Paupar* and *Pax6* promoters. We attempted to extract numerical curvature data from the 4CIn output to calculate statistical significance of the overlap between curated fragments and curvature points, but the software did not allow us access to the necessary information (genomic coordinates and curvature locations).

We agree with the reviewer that the statement “most curated fragments are located at curvature points on the chromatin fibre and appear to be orientated towards the Paupar-Pax6 promoters” is rather subjective and have therefore modified the text to state that “visual inspection suggests that most curated fragments are located at curvature points on the chromatin fibre and appear to be orientated towards the Paupar-Pax6 promoters”. The 3D model of chromatin architecture was produced to enable visual examination of the complex NG Capture-C sequencing datasets and facilitate interpretation.

3. The authors show that the induction of the constitutively active S33Y b-catenin leads to loss of Paupar and Pax6 expression. How does the expression of Tcf7L2 change? If Tcf7L2 drives loss of Paupar and Pax6, we would also expect loss of Tcf7L2 upon induction of S33Y.

We now include RT-qPCR experimental data showing that BcatS33Y over-expression does indeed lead to loss of Tcf7L2 as predicted with a concomitant down-regulation of *Paupar* and *Pax6* (Fig 3C). This is consistent with our observation that Tcf7L2 regulates Paupar-Pax6 co-expression in response to Wnt signalling.

4. The authors show that BMP4 induction causes an increase in Tcf7l2, Paupar, and Pax6 expression. These results are inconsistent with the Wnt3a induction data. That is, if Wnt3a activates BMP4 (as mentioned in the Szemes reference), then one would expect that induction of Wnt3a would phenocopy induction of BMP4. Instead, the data presented show Wnt3a and BMP4 have opposite effects.

Whilst the Szemes study showed that Wnt3a treatment activates Bmp4 expression in human SK-N-BE(2)-C cells Wnt signalling is known to exert context specific effects on target gene expression in neuroblastoma. As recombinant BMP4 upregulated Tcf7L2 (Fig 4E) and Wnt3a treatment reduced Tcf7L2 (Fig 4D) in N2A cells, we hypothesized that Wnt3a would also downregulate Bmp4. We tested this using RT-qPCR but could not detect Bmp4 expression above background levels in these cells. This suggests that Tcf7L2 upregulation in response to Wnt3a treatment is independent of Bmp4 induction in N2A cells and likely explains why Wnt3a and BMP4 have opposite effects. Nevertheless, the key finding of this set of experiments is that any changes in *Tcf7L2* levels in response to the different treatments are accompanied by reciprocal downstream alterations in *Paupar* and *Pax6* expression. These results are all consistent with a model in which Wnt signalling acts through *Tcf7l2* to co-ordinately regulate both *Paupar* and *Pax6* expression in neural cells.

5. The authors suggest that CRE2 and CRE5 are transcriptional repressors while CRE3 is an enhancer. The authors should perform further experiments to confirm this hypothesis. One possibility is to perform reporter assays in wild-type sequences compared to sequences with Tcf7l2 binding sites abolished.

We used CRISPRi to block CRE activity and determine regulatory element function within the context of endogenous chromatin, as opposed to utilising CRE reporter assays in which the DNA sequences are cloned into a reporter plasmid. We extended these experiments to annotate the function of all four ChIP-defined TCF7L2 bound candidate CREs (CRE1, CRE2, CRE3, CRE5) as well as two nearby non-TCF7L2 motif containing control sequences within the *Paupar*-*Pax6* regulatory region as suggested by Reviewer #2 (Fig 4C).

We found that blocking CRE2 and CRE5 function specifically increased, whilst blocking CRE3 specifically decreased, both Paupar and Pax6 expression. dCas9-KRAB recruitment to CRE1 and two nearby regions within the *Paupar*-*Pax6* regulatory region that do not contain TCF7L2 motifs did not lead to coordinated changes in *Paupar-Pax6* expression. These additional experiments show that the effect of disrupting CRE2, CRE3 and CRE5 is sequence specific and thus provide additional evidence in support of our hypothesis that CRE2 and CRE5 are transcriptional repressors while CRE3 is an enhancer.

6. The authors present statistical evidence that the Pax6 region exhibits asymmetry in chromatin architecture. It will be important to confirm this observation with independent experiments.

Some studies investigating long distant CRE-promoter looping interactions have used 3D-FISH to corroborate chromosome conformation capture (3C) based methods and the results are largely consistent. However, the resolution of 3D-FISH imaging is not sufficient to identify cell type differences between short-range chromatin interactions (all located +/- 50 kb from each viewpoint) that were identified in this study. We therefore were unable to apply this approach to independently confirm our findings.

However, our NG Capture-C data identified specific looping interactions between the previously defined Sox2 ESC enhancer located within the Peril locus and the Sox2 promoter (Supplemental S1 Fig). As expected, these interactions were present in ESCs but not in neurons showing that NG Capture-C can identify cell type differences in chromatin interactions such as the Pax6 promoter asymmetry that we observed. The Sox2 results represents robust validation of the NG-Capture C approach.

7. The authors previously showed (Vance et al, EMBO J, 2014) that loss of Paupar causes an increase in Pax6 expression (ie, they are anti-correlated). In contrast, all the perturbation experiments performed by the authors show that changes in Paupar expression are correlated with changes in Pax6. The authors need to reconcile these differences.

Thank you for highlighting this and we have now performed additional experiments and analysis to reconcile these differences.

Our previous work showed that depletion of *Paupar* using shRNA transfection led to a dose-dependent increase in *Pax6* expression after 3 days (PMID: 24488179). This suggests that *Paupar* represses *Pax6* transcription in N2A cells. However, this interpretation is complicated by the fact PAX6 binds its own promoter and can negatively regulate its own expression as shown in PMID: 17202185. Moreover, intersection of our previously determined *Paupar* genome-wide binding maps with microarray analysis of *Paupar* mediated gene expression changes identified 242 genes that are directly bound and regulated by *Paupar* in N2A cells (PMID: 24488179). Classification of up- and down-regulated gene expression changes showed that *Paupar* primarily activates the expression of its direct transcriptional target genes. This suggests that *Paupar* may also act to directly activate *Pax6* expression and that the observed increase in *Pax6* upon *Paupar* depletion may be due to de-repression of Pax6 negative feedback. This is consistent with our finding that the *Paupar* locus has enhancer-like chromatin marks.

To investigate the transcriptional regulatory function of *Paupar* further we tested whether induced proximity of the *Paupar* transcript regulates *Pax6* promoter activity using reporter assays. To do this, MS2-tagged *Paupar* was recruited to UAS sites upstream of a *Pax6* promoter reporter using a Gal4-MS2 phage coat protein (MCP) fusion protein in a transient transfection assay (Fig 4D). This resulted in a statistically significant (*P* = 0.006) 60% increase in *Pax6* promoter activity. Removal of Gal4-MCP from the assay, so that *Paupar* was no longer recruited to the reporter, led to a significant reduction in the *Paupar* transcriptional response whilst expression of Gal4-MCP fusion protein on its own had no effect on *Pax6* promoter activity (Fig 4D), confirming the specificity of the assay.

Considered together with the results in PMID: 17202185 showing PAX6 negative feedback, our data are consistent with a model in which *Paupar* and *Pax6* expression is coordinated and that the *Paupar* transcript up-regulates *Pax6* levels whilst PAX6 protein fine tunes its own expression through a negative auto-regulatory feedback loop.

Reviewer #2: In this manuscript entitled “Chromatin interaction maps identify Wnt responsive cis-regulatory elements coordinating Paupar-Pax6 expression in neuronal cells” Pavlaki and colleagues address the co-regulation of the lncRNA Paupar and its neighboring Px6 gene in the developing neural system.

The authors perform capture-c using either Paupar’s or the two main Pax6 promoters as anchors with the aim of identifying new regulatory sequences (either unique to each promoter or shared) that coordinate expression of the locus. The majority of interactions identified seems unique for each promoter type. Next, the authors focus on the N2A neuroblastoma cell line data and curate a set of 42 interactions to be further characterized. Using motif analysis, TCF7L2 (effector of the Wnt-Beta catenin pathway) is identified as a potential regulator of these cis-regulatory elements. After confirming binding with ChIP assays, the authors deplete the transcription factor to show this causes some reduction of Paupar and Pax6 expression. Next, dCas9-mediated repression of select elements is used to demonstrate that TCF7L2-bound CREs can either prompt repression or activation of Paupar and Pax6 in a coordinated fashion.

The work presented is highly correlative and falls short of demonstrating a key role for TCF7L2, here are the main weaknesses:

-the motif analysis leading to TCF7L2 needs a better explanation: we could not retrieve the location of the BiFa tool and the reference indicated does not provide any help in that regard. Which other motifs were identified? What convinced the authors that TCF7L2 was the most significant? What did they use as a control dataset?

We apologise for the lack of clarity and present a more detailed description of the motif analysis, including the correct reference describing the motif discovery tool (PMID: 21047506). This contains a description of the position frequency matrices (PFM) scanning procedure and model used to evaluate binding site matches against background.

To investigate the transcription factors controlling *Paupar-Pax6* co-expression in the brain we used this tool to search for transcription factor PFMs within the N2A cell curated fragment dataset that were calculated as having a high likelihood of factor binding (as described in PMID: 21047506). This identified 269 high scoring transcription factor motifs that occur more than 10 times within the set of curated fragments (listed in Table S7).

We selected five of these transcription factors (Klf16, Tcf7L2, Arid3a, Mash1 (Ascl1) and Sox6) with known functions in neuronal development to test for roles in *Paupar*-*Pax6* expression control.

To do this, we transfected N2A cells with specific endoribonuclease-prepared pools of siRNAs (esiRNAs) targeting these transcription factors and measured changes in *Paupar* and *Pax6* expression using RT-qPCR. The results showed that depletion of Tcf7L2 and Mash1 (Ascl1) led to a significant decrease in both *Paupar* and *Pax6* (Fig 3B), suggesting that Tcf7L2 and Mash1 are involved in coordinating *Paupar* and *Pax6* activation in neuronal cells. We also discovered that silencing Klf16, Sox6 and Arid3a led to a significant increase in *Paupar* but no significant change in *Pax6* (Fig 3A), indicating that *Paupar-Pax6* expression control can also be uncoupled and that these factors act to selectively silence *Paupar.*

We selected Tcf7L2 for further investigation as it is a major effector of the Wnt/b-catenin key neuro-developmental signalling pathway, an important regulator of chromatin structure, and our results suggest that it is involved in coordinating *Paupar* and *Pax6* co-expression control in neuronal cells.

-the robustness of TCF7L2 binding should be assessed unbiasedly by ChIP-seq or CUT&RUN. ChIP-qPCR is not an acceptable standard anymore and fold enrichment over IgG can be very misleading.

We performed ChIP-qPCR in this study, as opposed to ChIP-seq, as we did not want to map TCF7L2 genome-wide occupancy. Instead, we sought to test a specific hypothesis (based on our motif analysis and TCF7L2 perturbation experiments) to determine TCF7L2 binding at a set of predicted motifs within the *Paupar*-*Pax6* gene regulatory region. We don’t agree that ChIP-qPCR is not an acceptable standard anymore and this approach is still very widely used to investigate TF binding at defined regions of the genome.

Taken together, the results from our ChIP, CRISPRi functional annotation of TCF7L2 bound CREs, and gene expression profiling of Tcf7L2 knockdown and signalling pathway manipulation experiments, all provide strong evidence that TCF7L2 binds and directly co-ordinates *Paupar*-*Pax6* expression in N2A cells.

-the dCas9 approach needs a more thorough control set. In order to demonstrate that the effect of disrupting CRE2, CRE3 and CRE5 is sequence specific (and not the aftermath of altering the chromatin landscape at the whole region) the authors need to design 2 or 3 control gRNAs targeting non CRE elements within the Paupar-Pax6 regulatory region.

We extended the CRISPRi experiments to annotate the function of all four ChIP-defined TCF7L2 bound candidate CREs (CRE1, CRE2, CRE3, CRE5) as well as two nearby non-TCF7L2 motif containing control sequences within the *Paupar*-*Pax6* regulatory region as suggested to validate the specificity of the CRISPRi transcriptional response.

We found that blocking CRE2 and CRE5 function specifically increased, whilst blocking CRE3 specifically decreased, both Paupar and Pax6 expression. dCas9-KRAB recruitment to CRE1 and two nearby regions within the *Paupar*-*Pax6* regulatory region that do not contain TCF7L2 motifs did not lead to coordinated changes in *Paupar-Pax6* expression. These additional experiments show that the effect of disrupting CRE2, CRE3 and CRE5 is sequence specific and thus provide additional evidence in support of our hypothesis that CRE2 and CRE5 are transcriptional repressors while CRE3 is an enhancer.

Minor:

Figure 2 could be a supplemental figure as it is a mere display of technical execution without pointing out any specific interaction.

We have merged Fig 1 and Fig 2 together in the revised manuscript.
